# La-related protein 1 (LARP1) binds the mRNA cap, blocking eIF4F assembly on TOP mRNAs

**DOI:** 10.7554/eLife.24146

**Published:** 2017-04-07

**Authors:** Roni M Lahr, Bruno D Fonseca, Gabrielle E Ciotti, Hiba A Al-Ashtal, Jian-Jun Jia, Marius R Niklaus, Sarah P Blagden, Tommy Alain, Andrea J Berman

**Affiliations:** 1 Department of Biological Sciences, University of Pittsburgh, Pittsburgh, United States; 2 Children’s Hospital of Eastern Ontario Research Institute, Ottawa, Canada; 3 Department of Oncology, University of Oxford, Oxford, United Kingdom; Johns Hopkins University, United States

**Keywords:** TOP mRNAs, 5' cap, La-related protein 1, eIF4E, RNA binding protein, X-ray crystallography, *E. coli*, Human

## Abstract

The 5’terminal oligopyrimidine (5’TOP) motif is a *cis*-regulatory RNA element located immediately downstream of the 7-methylguanosine [m^7^G] cap of TOP mRNAs, which encode ribosomal proteins and translation factors. In eukaryotes, this motif coordinates the synchronous and stoichiometric expression of the protein components of the translation machinery. La-related protein 1 (LARP1) binds TOP mRNAs, regulating their stability and translation. We present crystal structures of the human LARP1 DM15 region in complex with a 5’TOP motif, a cap analog (m^7^GTP), and a capped cytidine (m^7^GpppC), resolved to 2.6, 1.8 and 1.7 Å, respectively. Our binding, competition, and immunoprecipitation data corroborate and elaborate on the mechanism of 5’TOP motif binding by LARP1. We show that LARP1 directly binds the cap and adjacent 5’TOP motif of TOP mRNAs, effectively impeding access of eIF4E to the cap and preventing eIF4F assembly. Thus, LARP1 is a specialized TOP mRNA cap-binding protein that controls ribosome biogenesis.

**DOI:** http://dx.doi.org/10.7554/eLife.24146.001

## Introduction

Ribosome biogenesis is a complex and energetically costly process for the cell. Eukaryotic cells exert precise temporal and stoichiometric control over ribosomal protein expression to ensure the production of functional ribosomes meets the growth demands of the cell. Multicellular organisms regulate ribosome biogenesis post-transcriptionally, at the level of mRNA translation (Meyuhas and Kahan, 2015; Perry, 2007). All transcripts encoding ribosomal proteins, most translation factors, and some RNA-binding proteins carry a *cis-*regulatory RNA element termed the 5’terminal oligopyrimidine (5’TOP) motif comprising an invariant 5’-cytidine followed by an uninterrupted tract of 4–14 pyrimidine nucleotides and preceded by the 7-methylguanosine triphosphate (m^7^Gppp) cap (Fonseca et al., 2014; Iadevaia et al., 2008; Meyuhas and Kahan, 2015; Levy et al., 1991). This motif is essential for translation regulation of TOP mRNAs (Biberman and Meyuhas, 1999; Avni et al., 1994; Meyuhas and Kahan, 2015).

The mammalian target of rapamycin complex 1 (mTORC1) signaling pathway plays a prominent role in the control of TOP mRNA translation (Fonseca et al., 2014; Hsieh et al., 2012; Thoreen et al., 2012). In response to provision of nutrients (such as amino acids, growth factors, glucose, or oxygen), the growth-associated kinase complex mTORC1 boosts the production of components of the translation machinery encoded by TOP mRNAs (Meyuhas and Kahan, 2015; Chantranupong et al., 2015). Pharmacological inhibition of mTORC1 results in the pronounced repression of TOP mRNA translation (Terada et al., 1994; Jefferies et al., 1994; Thoreen et al., 2012; Hsieh et al., 2012).

Genome-wide ribosome profiling studies demonstrate that TOP mRNAs are the class of transcripts that are most sensitive to translation suppression by mTOR inhibitors (Thoreen et al., 2012; Hsieh et al., 2012). TOP mRNA translation has been the subject of intense study and several mechanisms have been proposed for its regulation by mTORC1 to date. Most recently, the mRNA cap-binding protein, eIF4E, and its repressor proteins, 4E-BP1 and 4E-BP2, have been linked to the control of TOP mRNA translation downstream of mTORC1 (Hsieh et al., 2012; Thoreen, 2017; Thoreen et al., 2012), but the exact contribution of 4E-BPs to the control of TOP mRNA translation remains a point of contention (Miloslavski et al., 2014). While 4E-BPs and many other candidates have been linked to the control of TOP mRNA translation (discussed in detail in (Meyuhas and Kahan, 2015)), the identity of the *bona fide* trans-acting factor that represses TOP mRNA translation downstream of mTORC1 remained enigmatic.

Recently, we and others (Fonseca et al., 2015; Tcherkezian et al., 2014) identified La-related protein 1 (LARP1) as a novel downstream target of mTORC1 and proposed that it plays an important role in the translation of TOP mRNAs. Specifically, we have shown that LARP1 associates with the regulatory-associated protein of mTOR (RAPTOR) when mTORC1 is active. Upon mTORC1 inhibition, a cellular state that is associated with TOP mRNA translation repression, we demonstrated that LARP1 dissociates from mTORC1 and binds to the 5’TOP motif of TOP mRNAs (Fonseca et al., 2015). We have also shown that LARP1 associates with the 5’TOP motif via a LARP1 family-specific ‘DM15 region’ located within its C-terminus (Lahr et al., 2015). In the present study, we confirm a direct association between the DM15 region and the 5’TOP motif.

Most importantly, our crystallographic data revealed an unexpected, but seminal role for the DM15 region of LARP1 in specialized cap-binding of TOP mRNAs. We show that the DM15 region of LARP1 specifically binds the 7-methylguanosine 5’−5’ triphosphate (m^7^Gppp) moiety and the invariant first cytidine of TOP mRNAs. Biochemical analyses reveal that LARP1 selectively prevents the binding of eIF4E to the m^7^Gppp cap to block the assembly of the eIF4F complex on TOP mRNAs. These important findings highlight a previously unrecognized dynamic interplay between LARP1 and eIF4F in the control of TOP mRNA translation and reconcile earlier, seemingly contradictory models of TOP mRNA translation control.

## Results and discussion

**Figure 1.**
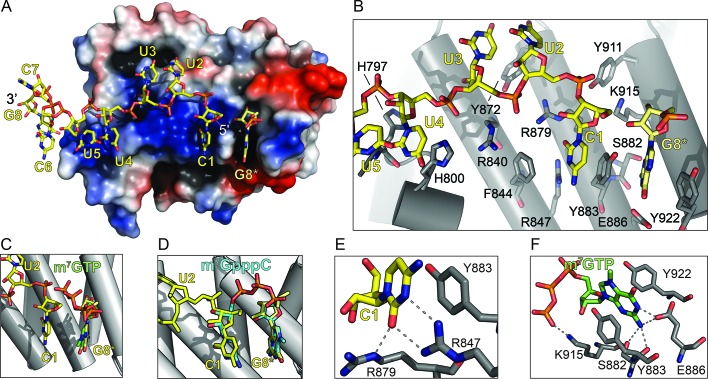
The LARP1 DM15 region recognizes the 7-methylguanosine cap and invariant 5’cytidine of TOP mRNAs. (**A**) Protein surface representation is colored according to electrostatic potential (−74 kEV, red; 74 kEV, blue). (**B**) Zoomed view of the DM15 RNA binding site. (**C**) Superimposition of DM15 bound to RNA and bound to cap analog, m^7^GTP. (**D**) Superimposition of DM15 bound to RNA and bound to m^7^GpppC. (**E–F**) Zoomed views of the specific recognition of C1 (**E**) and m^7^GTP (**F**). Potential hydrogen bonds indicated by dotted lines. **DOI:** http://dx.doi.org/10.7554/eLife.24146.002

To better understand how LARP1 engages the 5’TOP motif and controls TOP mRNA translation, we determined the 2.6 Å resolution X-ray crystal structure of the DM15 region (DM15) of human LARP1 bound to an RNA oligonucleotide spanning a segment of the 5’TOP motif of ribosomal protein S6 (RPS6) mRNA. We selected nucleotides 4–11 of the 42-nucleotide TOP sequence of RPS6 for co-crystallization experiments (5’-CCUCUUUUCCG-3’; the sequence used in co-crystallization experiments is underlined). The sequence and length choice was informed by the dimensions of the identified RNA binding site in the structure of *apo* DM15 and the results of nuclease protection assays performed on a complex of DM15 with the first 42 nucleotides of the RPS6 mRNA (Lahr et al., 2015). Importantly, despite excluding the first three nucleotides of the biological RPS6 TOP sequence, the sequence chosen for crystallization fits the definition of a TOP motif: a short stretch of pyrimidines preceded by a cytidine and succeeded by a guanosine (Meyuhas and Kahan, 2015). As anticipated, based on the negatively-charged phosphate backbone of the RNA, the resulting RNA-bound structure of DM15 revealed that the 5’TOP sequence binds to the highly conserved, positively charged surface of the three tandem helix-turn-helix HEAT-like repeats of DM15, termed A, B, and C (Figure 1A, Figure 1—figure supplement 1, Table 1) .

**Figure 1—figure supplement 1.**
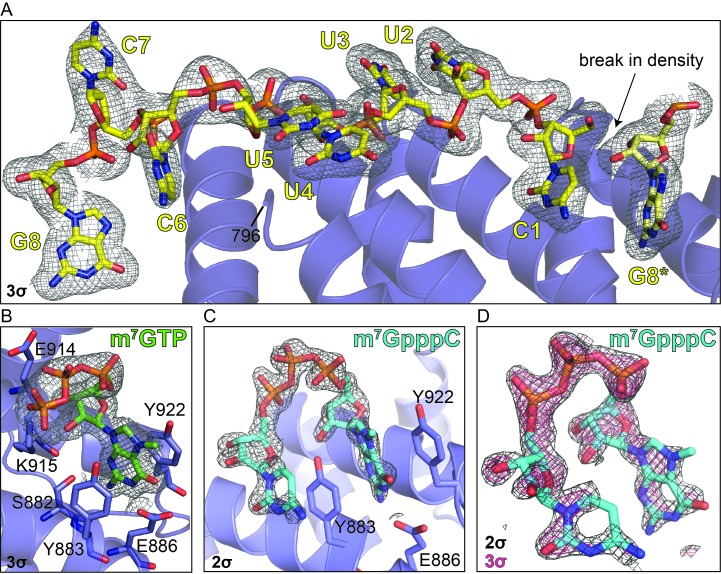
Electron density reveals RNA, cap analog, and m^7^GpppC bind in the same location on the conserved surface of the DM15 region of LARP1. Composite omit maps carved around the (**A**) RNA (3σ), (**B**) m^7^GTP cap analog (3σ), and (**C**) m^7^GpppC dinucleotide (2σ). (**D**) Composite omit map carved around the m^7^GpppC dinucleotide at 2σ (grey) and 3σ (magenta) for comparison. **DOI:** http://dx.doi.org/10.7554/eLife.24146.003

**Figure 1—figure supplement 2.**
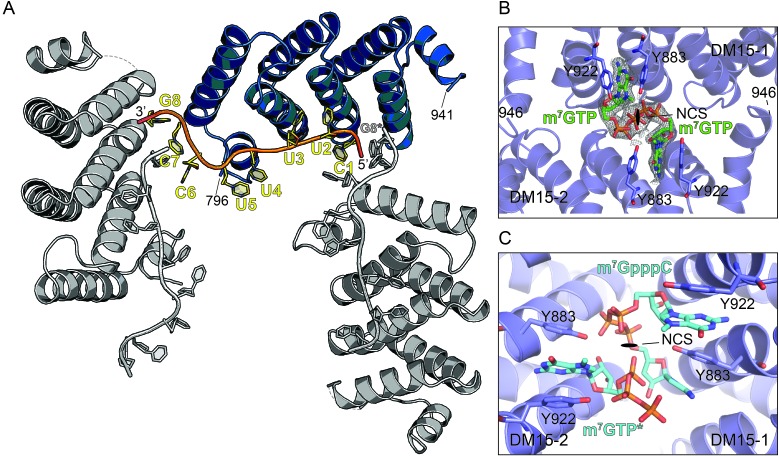
The DM15 region of LARP1 recognizes a guanosine. (**A**) Three neighboring unit cells from the DM15-RNA co-crystal are shown. The protein monomer colored in blue interacts with two molecules of RNA: one from its unit cell and one originating in the unit cell on the right. This places a guanosine nucleotide 5’ to the TOP motif in the binding site of the blue DM15 region. (**B**) Two non-crystallographic symmetry mates from the DM15-m^7^GTP co-crystal show the guanine of the cap analog binds the same place as the G8* residue binds in the DM15-RNA co-crystal (**A**). (**C**) Two non-crystallographic symmetry mates from the DM15-m^7^GpppC co-crystal structure reveal that the m^7^G and C moieties bind in the same place as the G8* and C1 residues in the DM15-RNA co-crystal, respectively. In the other non-crystallographic symmetry-mate shown, only m^7^GTP is modeled because only one dinucleotide can bind per asymmetric unit since the inverted triphosphate linkage sits on the NCS 2-fold axis (see Figure 1—figure supplement 5). **DOI:** http://dx.doi.org/10.7554/eLife.24146.004

**Figure 1—figure supplement 3.**
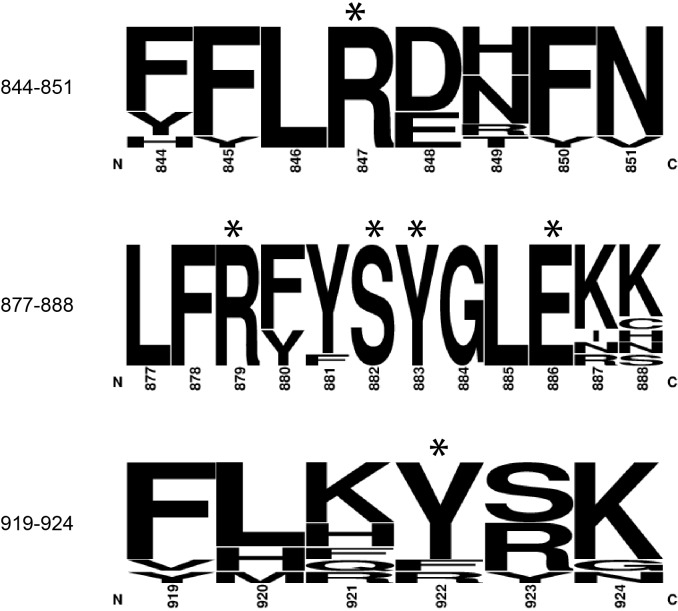
The amino acids of the DM15 region that directly bind G8*, C1, and m^7^GTP are nearly 100% conserved. Frequency plots were generated from amino acid sequences of LARP1 from *H. sapiens* (NP_056130.2), *M. musculus* (NP_082727.1), *D. rerio* (XP_001920902.3), *X. laevis* (NP_001089363.1)*, D. melanogaster* (NP_524998.1)*, C. elegans* (NP_001040867.2)*, A. thaliana* (NP_001190354.1), *O. sativa* (XP_015621184.1), *B. dendrobatidis* (XP_006676827.1), and *M. brevicollis* (XP_001744631.1). Asterisks indicate amino acids shown in Figure 1. Numbering is based on the DM15 region construct cloned from LARP1 isoform 2 (NP_056130.2). **DOI:** http://dx.doi.org/10.7554/eLife.24146.005

**Figure 1—figure supplement 4.**
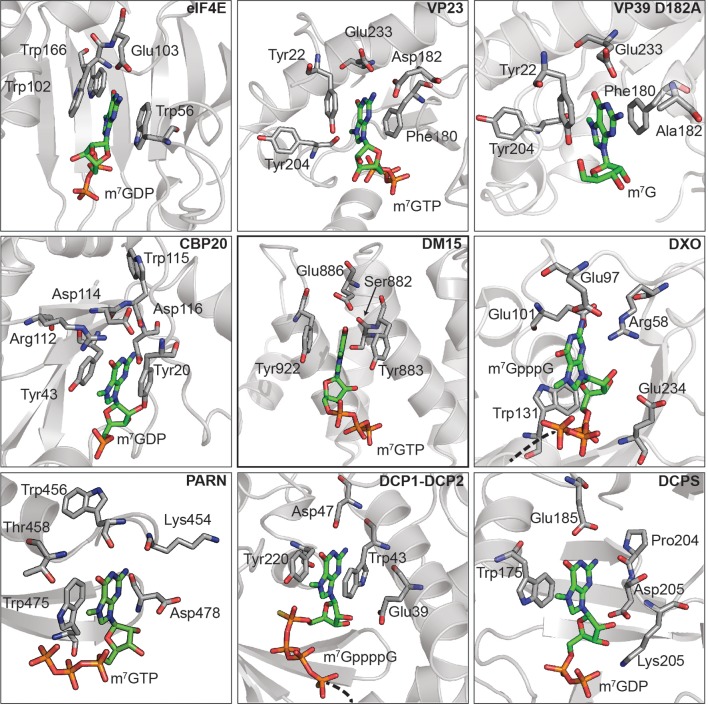
The DM15 region of LARP1 has evolved a unique fold to support a canonical cap-binding pocket. The amino acids responsible for cap recognition in each of the proteins are shown as white sticks and the cap analog is shown as green sticks. PDBs used to generate each panel: eIF4E (1EJ1), VP23 (1AV6), VP39 D182A (4DCG), CBP20 (1H2T), LARP1 DM15 (5V4R), DXO (4J7N), PARN (3CTR), DCP1-DCP2 (5KQ4), DCPS (1XMM). **DOI:** http://dx.doi.org/10.7554/eLife.24146.006

**Figure 1—figure supplement 5.**
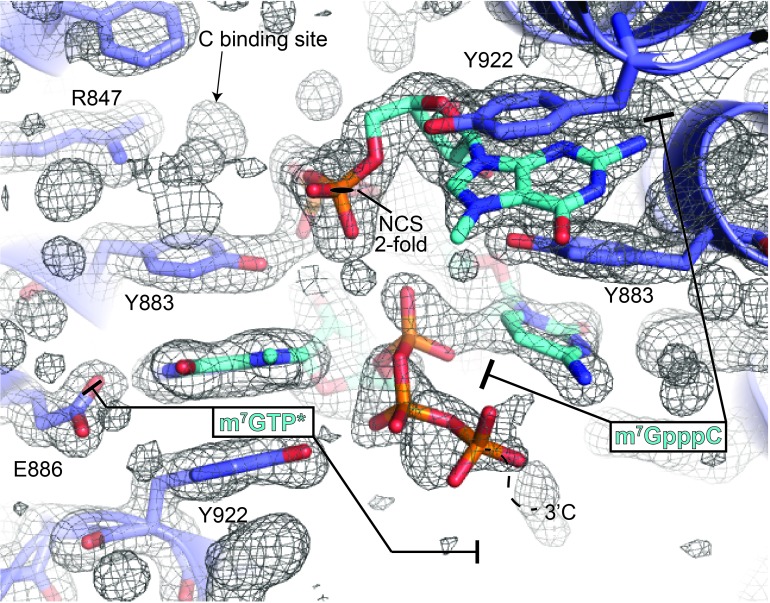
The m^7^GpppC-DM15 co-crystal non-crystallographic symmetry reveals one ordered dinucleotide and one partially ordered dinucleotide. The composite omit map shown is contoured at 1σ and reveals an ordered m^7^GpppC dinucleotide bound to the DM15 NCS-mate on the right. The DM15 NCS-mate on the left binds the m^7^G in the G-binding pocket, while the triphosphate moiety is ordered and facing away from the C-binding pocket (m^7^GTP*). Presumably, the density for the cytidine is mostly disordered: based on the path of the m^7^G triphosphate moiety (dotted line and 3'C label), the cytidine is most likely in the solvent channel; density for the Watson-Crick face of a cytidine is visible in the C-binding site in this copy of DM15, but is presumably of very low occupancy. Based on this data and the data shown in Figure 1—figure supplement 2B, only one inverted triphosphate per asymmetric unit can be accommodated in the anion binding site that overlaps with the non-crystallographic 2-fold axis. **DOI:** http://dx.doi.org/10.7554/eLife.24146.007

**Table 1.** X-ray data collection and refinement statistics. **DOI:** http://dx.doi.org/10.7554/eLife.24146.008

Data collection	m^7^GTP	RNA-bound	m^7^GpppC
Space group	P2_1_	I4	P2_1_
Unit cell dimensions			
a, b, c (Å)	48.177, 60.163, 60.609	107.095, 107.095, 29.113	47.959, 59.962, 60.321
β	101.283	90	100.653
Resolution (Å)	33.90–1.77	37.86–2.59	30.00–1.69
R_merge_ (%)	3.5 (27.7)	15.6 (37.1)	3.6 (12.5)
I/σ(I)	16.30 (2.1)	8.79 (1.36)	19.85 (8.8)
Completeness (%)	82 (73)	98.5 (87.1)	92.2 (93.2)
Redundancy	2.9 (1.5)	4.3 (2.1)	3.4 (2.8)
			

Refinement	m^7^GTP	RNA-bound	m^7^GpppC
Resolution (Å)	33.90–1.77	37.86–2.59	30.00–1.69
No. reflections	28525	5272	34328
Completeness (%)	82 (73)	98.5 (87.1)	92.2 (93.2)
R_work_/R_free_	17.50/20.80	20.69/23.51	18.10/20.30
RMSD bond angle (°)	1.43	1.19	0.89
RMSD bond length (Å)	0.012	0.015	0.010
Average B-factor	42.0	36.4	33.9
PDB ID	5V4R	5V7C	5V87

Fortuitously, the sequence of RNA chosen for co-crystallization experiments promoted additional crystal contacts, which revealed an unanticipated function for the DM15, discussed below. Co-crystallization of DM15 with part of the RPS6 5’TOP motif yielded a crystal lattice in which one protein molecule interacts with two molecules of RNA in the unit cell. A given protein molecule simultaneously binds nucleotides 1–5 of one RNA molecule and the 3’ guanosine (G8) of the RNA bound to the protein in the neighboring unit cell (Figure 1—figure supplement 2A). G8 sits ‘upstream’ or 5’ to nucleotides 1–5; we denote the 3’ terminal G derived from the RNA strand originating in the neighboring unit cell as G8* to indicate its discontinuity from the polypyrimidine motif in nucleotides 1 to 5.

Our observations of (1) a binding pocket complementary to the size and shape of a guanosine that (2) specifically recognizes its Watson-Crick face, and (3) sits upstream of the 5’ terminal cytidine of the bound TOP RNA led us to hypothesize that DM15 binds the cap of TOP mRNAs. To test this, we determined the co-crystal structure of DM15 bound to the cap analog 7-methylguanosine triphosphate (m^7^GTP) to 1.8 Å resolution. m^7^GTP binds the same pocket as G8* in the DM15-RNA co-crystal (Figure 1C, Figure 1—figure supplements 1B and 2B). Lysine-915 stabilizes the γ-phosphate of m^7^GTP (Figure 1F). Interestingly, the γ-phosphate of m^7^GTP aligns with the position of the bound sulfate ion that crystallized at the non-crystallographic dimer interface in the structure of the *apo* DM15 region (Lahr et al., 2015).

Residues from each of the conserved DM15 repeats (A, B, C) participate in the recognition of the invariant cytidine in the first position of TOP mRNAs (C1) and the guanosine (G8*) 5’ to it (Figure 1B–D). All of the amino acids that interact directly with the RNA are nearly 100% conserved from worms to mammals and plants (Figure 1—figure supplement 3) (Bousquet-Antonelli and Deragon, 2009; Lahr et al., 2015). Amino acids R840, R879, and H800 define the path of the RNA by aligning it on the surface of DM15 through ionic and hydrogen bonding interactions with the phosphate backbone. Amino acids Y922, Y883, and F844 stabilize the nucleobases of G8* and C1 through stacking interactions. Sequence-specific recognition of G8* through its Watson-Crick face is accomplished by hydrogen bonds with E886 and S882. Arginines 847 and 879 specifically recognize C1 (Bousquet-Antonelli and Deragon, 2009; Lahr et al., 2015). The position of R879 forces U2 to flip away from stacking with C1. Instead, U3 stacks on U2. The base of U4 flips back toward C1 to stack on H800, while the O2 on its Watson-Crick face hydrogen bonds with the backbone carbonyl of H797.

While the α-helical nature of DM15 is unique among cap-binding proteins, its cap recognition pocket exhibits the canonical architecture (Figure 1—figure supplement 4). The DM15 region of LARP1 stabilizes the nucleobase of m^7^GTP between two conserved aromatic amino acids, Y883 and Y922. In addition, E886 hydrogen bonds with the Watson-Crick face of the guanosine moiety, using acidic side chains for this recognition in a mechanism reminiscent to that of other cap-binding proteins (Figure 1—figure supplement 4, CBP20). The methyl group points away from the core of the DM15 region in a manner similar to that observed in the nuclear cap-binding complex; it is therefore likely that other regions of LARP1 outside of the crystallized construct interact with the methyl group at position 7 and the 5’−5’ triphosphate linkage connecting the m^7^Gppp moiety and the first cytidine nucleotide of the TOP motif.

The conservation of the residues interacting with m^7^GTP in addition to the nucleotide-specific recognition of G8* and C1 from the DM15-RNA and DM15-m^7^GTP co-crystal structures suggest the DM15 region of LARP1 specifically recognizes the m^7^GpppC motif. To test this hypothesis, we determined the crystal structure of DM15 bound to m^7^GpppC dinucleotide. Indeed, this dinucleotide binds the DM15 region in the cap- and C-binding pockets (Figure 1, Figure 1—figure supplements 1, 2 and 5).

**Figure 2.**
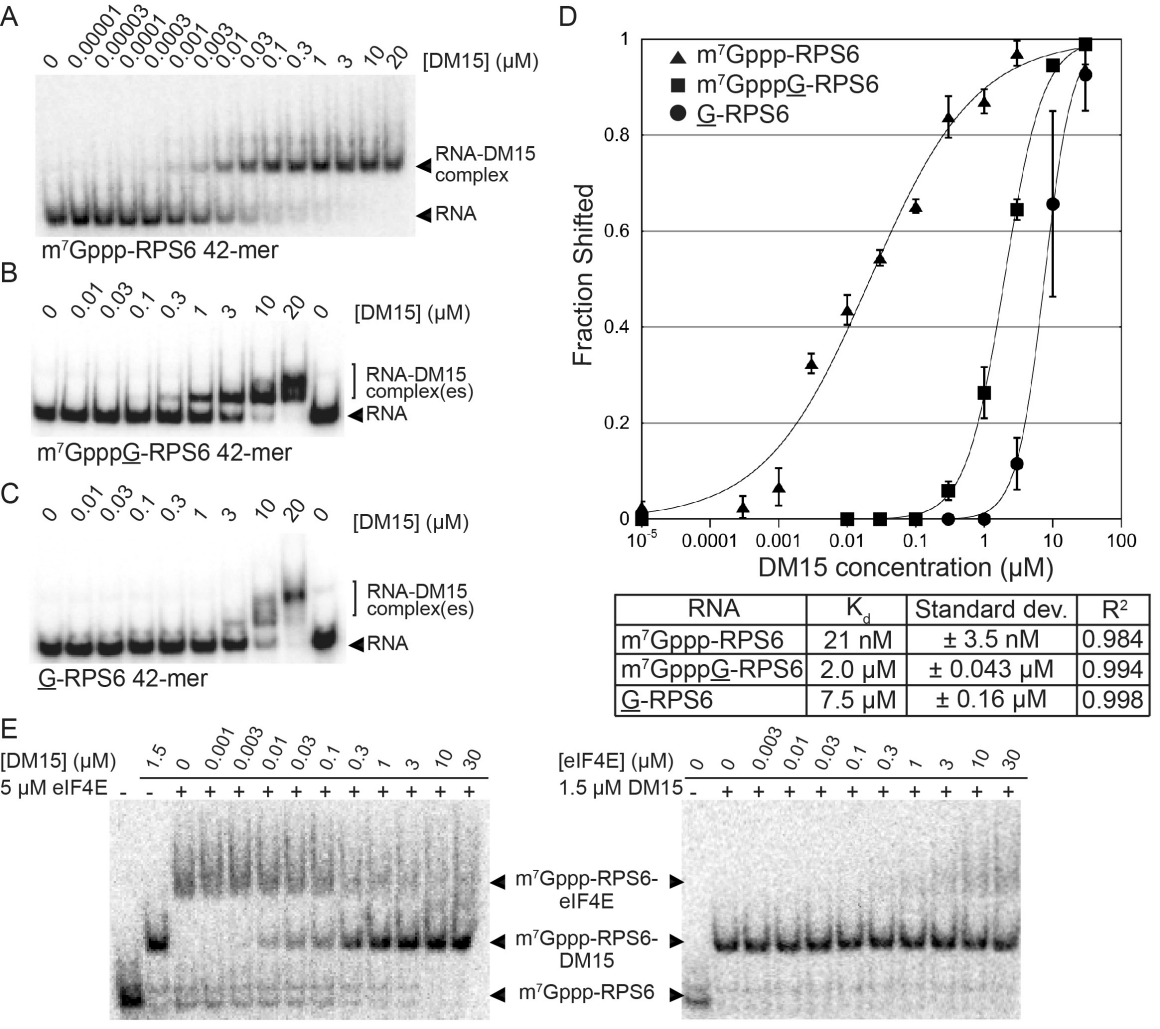
The LARP1 DM15 region recognizes capped TOP sequences and outcompetes eIF4E for their binding. (**A–C**) Electrophoretic mobility shift assays using the indicated RNA. (**D**) Quantitation of 3 replicate EMSAs; error bars represent standard deviation. (**E**) Competition assays analyzed by native gel electrophoresis using labeled m^7^Gppp-RPS6 as substrate with the indicated protein concentrations. **DOI:** http://dx.doi.org/10.7554/eLife.24146.009 10.7554/eLife.24146.010Figure 2—source data 1.Data for graphed EMSAs.**DOI:** http://dx.doi.org/10.7554/eLife.24146.010

In vitro biochemical RNA-binding assays analyzed by electrophoretic mobility shift corroborate our crystallographic observations. DM15 binds a 42-mer oligonucleotide of the 5’UTR of RPS6 containing an additional 5’G, effectively making it a non-5’TOP sequence, with an affinity of 7.5 μM; capping this substrate enhances affinity 3.8-fold (2.0 μM). Capping the TOP 42-mer RPS6 RNA substrate increases the affinity 360-fold (Figure 2; 21 nM) over the non-TOP substrate and 95-fold over the capped non-TOP substrate.

**Figure 2—figure supplement 1.**
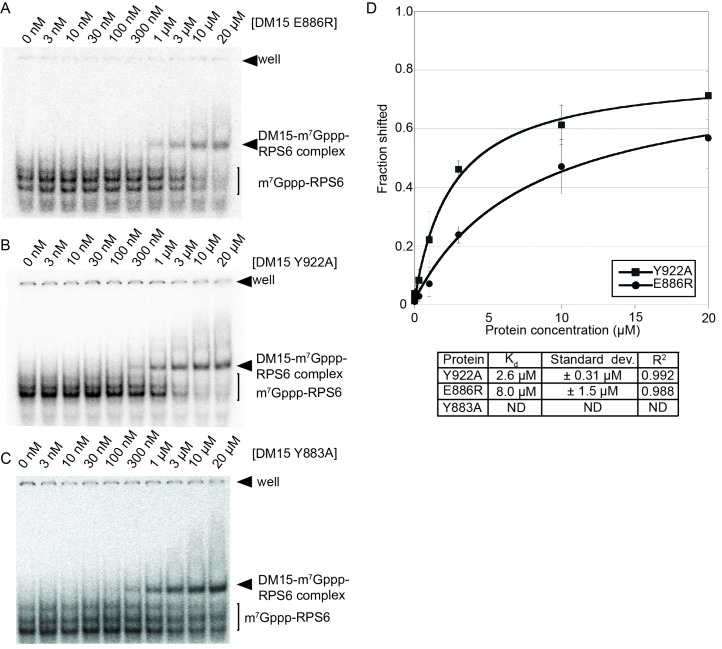
Mutation of amino acids that stabilize the cap and the RNA decreases the affinity of the DM15 region for capped TOP RNA. (**A–C**) EMSA of capped RPS6 42-mer 5’UTR with each indicated point mutant. (**D**) Quantification of three replicates of each indicated EMSA. ND, not determined. **DOI:** http://dx.doi.org/10.7554/eLife.24146.011

**Figure 2—figure supplement 2.**
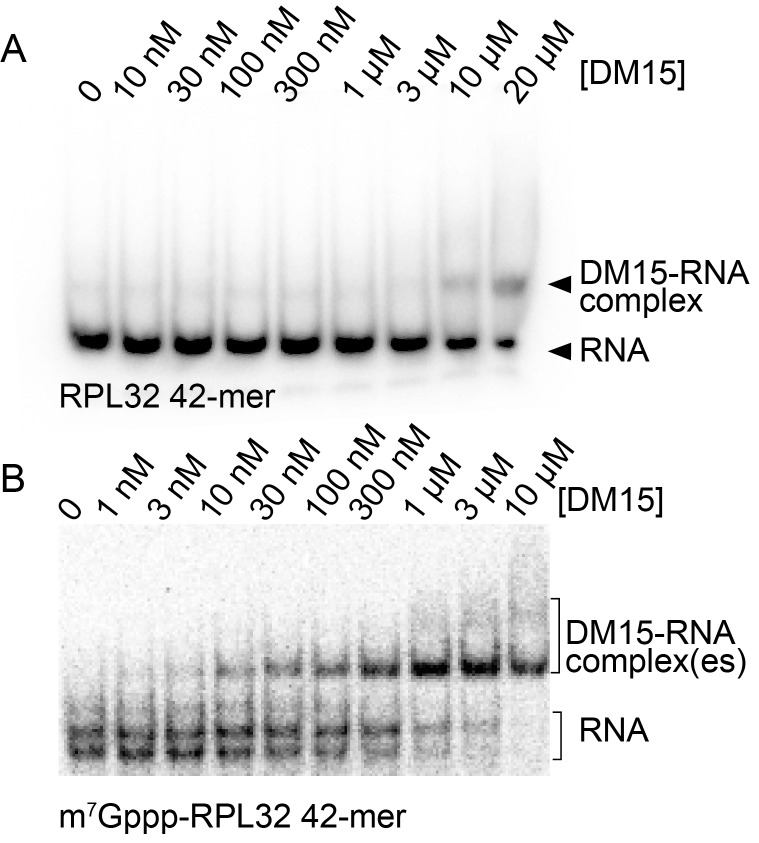
Capping another TOP sequence enhances the affinity of DM15 for the RNA. (**A**) EMSA of DM15 with an uncapped RNA representing the first 42 nucleotides of the 5’UTR of RPL32. (**B**) EMSA of DM15 with the capped version of the RNA from panel (**A**). **DOI:** http://dx.doi.org/10.7554/eLife.24146.012

**Figure 2—figure supplement 3.**
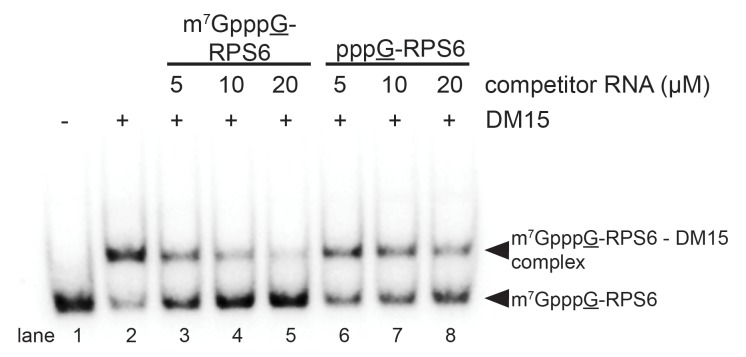
The interaction between the DM15 region of LARP1 and the cap moiety is specific. Competition assay challenging DM15 bound to labeled capped non-TOP RNA with capped (lanes 3–5) or uncapped (lanes 6–8) cold competitor RNA. **DOI:** http://dx.doi.org/10.7554/eLife.24146.013

**Figure 2—figure supplement 4.**
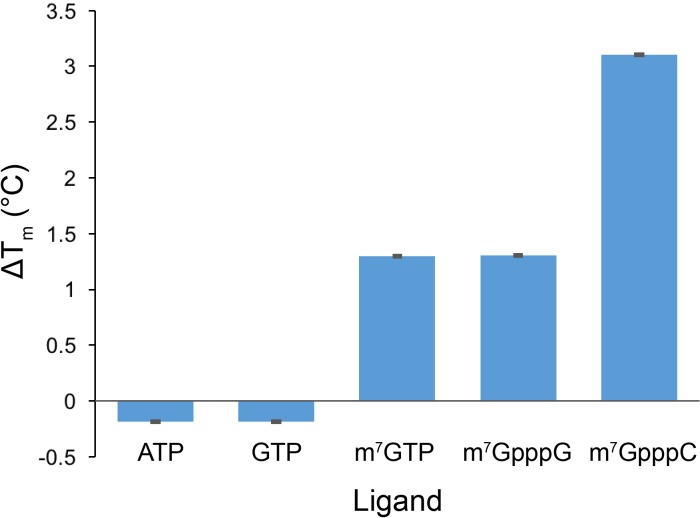
Cap analog, m^7^GTP, and m^7^GpppC stabilize the protein fold of the DM15 region of LARP1. Quantification of thermal shift assays conducted in the presence of GTP, m^7^GTP, ATP, m^7^GpppG, or m^7^GpppC; n = 3 for all assays; error bars show 1 standard deviation. **DOI:** http://dx.doi.org/10.7554/eLife.24146.014

**Figure 2—figure supplement 5.**
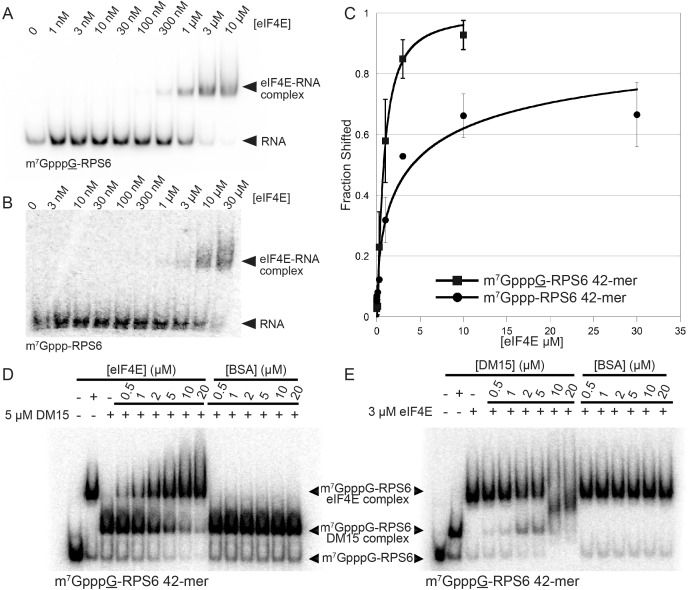
eIF4E binds non-TOP mRNAs and outcompetes the DM15 region of LARP1 for their binding. (**A–B**) EMSAs with the indicated substrates and increasing concentrations of recombinant human eIF4E. (**C**) Quantitation of EMSAs, n = 3. Error bars indicate standard deviation. (**D–E**) Competition assays for the capped non-TOP substrate. **DOI:** http://dx.doi.org/10.7554/eLife.24146.015

Mutating the residues in DM15 that stabilize the cap, E886, Y922, or Y883, decreases the affinity for capped TOP RNA 380-fold (8.0 μM), 124-fold (2.6 μM), and greater than ~950 fold (N.D.,>20 μM), respectively (Figure 2—figure supplement 1). Capping another 5’TOP sequence comprised of the first 42 nucleotides of the 5’UTR of RPL32 mRNA enhances the interaction of DM15 with this sequence (Figure 2—figure supplement 2). Further, competition assays in which capped mRNAs better displace capped DM15-bound sequences than uncapped mRNAs do, suggest the interaction of DM15 with the cap moiety is specific (Figure 2—figure supplement 3). Finally, thermal shift stability assays demonstrate the interaction of DM15 with m^7^GTP and m^7^GpppC is increasingly stabilizing to the DM15 fold (Figure 2—figure supplement 4); notably, the m^7^GpppG dinucleotide does not impart additional stability to DM15 over m^7^GTP. Together with the conservation of the residues interacting with m^7^GTP, the base-specific hydrogen bonding of the Watson-Crick faces of nucleobases of G8* and C1 in the DM15-RNA co-crystal structure and the recognition of the m^7^GpppC dinucleotide in the DM15-m^7^GpppC co-crystal structure, these data suggest the DM15 region of LARP1 specifically recognizes the m^7^GpppC motif characteristic of TOP mRNAs. This observation is particularly remarkable because the first cytdine is indispensable for repression of TOP mRNA translation (Levy et al., 1991). Interestingly, the first cytidine is also essential for LARP1 association with RPS6 and RPL32 mRNAs (Fonseca et al., 2015). Higher-order complexes and nonspecific binding of DM15 to the RNA oligonucleotide are greatly reduced in the capped-TOP substrate binding reaction (Figure 2), indicating that the m^7^GpppC motif might lock the register of DM15 binding. Furthermore, these data provide a molecular mechanism by which LARP1 differentiates TOP mRNAs from all other cellular mRNAs, most of which have a purine in the +1 position (Schibler et al., 1977; Schibler and Perry, 1977).

We hypothesized that if DM15 recognizes the caps of TOP mRNAs, it might compete for cap binding with eIF4E, the eukaryotic initiation factor required for canonical cap-dependent translation initiation (Sonenberg et al., 1978, 1979). To test this, we conducted competition assays between the human DM15 region and recombinant human eIF4E for capped oligonucleotides (Figure 2E). While displacement of DM15 from capped TOP RNA requires high-micromolar concentrations of eIF4E, low-nanomolar concentrations of DM15 are sufficient to displace eIF4E from this substrate. Interestingly, the opposite is true for capped non-TOP mRNA: eIF4E outcompetes DM15 for the non-TOP substrate as the pre-bound or competitor protein (Figure 2—figure supplement 3). These results are consistent with the preferred binding of eIF4E for m^7^GpppG (Kiraga-Motoszko et al., 2011; Thoreen et al., 2012; et al., Tamarkin-Ben-Harush et al., 2017) and with studies suggesting that quiescent cells and translation systems contain a factor that represses TOP mRNA translation even in the presence of excess eIF4E (Shama et al., 1995; Biberman and Meyuhas, 1999). Since most mRNAs have a purine in the +1 position (Schibler et al., 1977; Schibler and Perry, 1977), eIF4E is anticipated to stimulate their translation; by contrast, TOP mRNAs have an invariant C in the first position, suggesting that LARP1 would sequester them, thereby preventing cap-dependent translation initiation until a signaling event releases this repression. Of note, we show that the affinity of the DM15 region of LARP1 for TOP mRNAs is considerably higher than that of full-length eIF4E protein for this class of mRNAs, likely explaining why excess eIF4E alone is insufficient to upregulate the translation of TOP mRNAs (Shama et al., 1995).

The eIF4F complex (formed by the cap-binding protein eIF4E, the scaffolding protein eIF4G and the ATP-dependent RNA helicase eIF4A) has been linked to the control of TOP mRNA translation (Thoreen et al., 2012; Hsieh et al., 2012). Moreover, eIF4G co-precipitates TOP mRNAs in an mTORC1–dependent manner (Fonseca et al., 2015). Notably, the ability of TOP mRNAs to associate with eIF4G is markedly inhibited upon overexpression of LARP1 in mammalian cells (Fonseca et al., 2015), consistent with the idea that LARP1 competes with eIF4E and eIF4G for binding to TOP mRNAs. This notion is substantiated by the present findings that LARP1 blocks the access of eIF4E to m^7^Gppp-TOP mRNAs.

**Figure 3.**
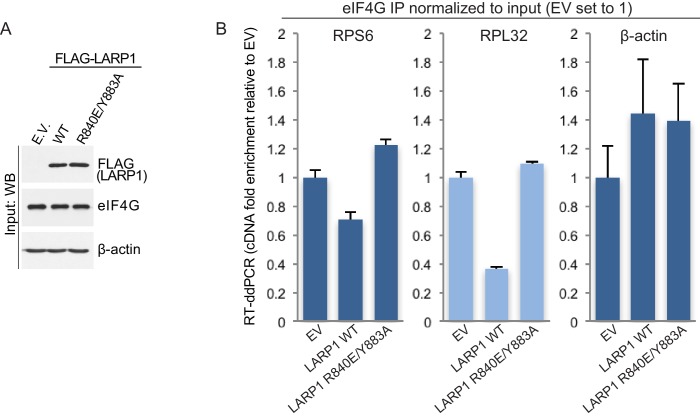
LARP1 prevents eIF4F assembly on 5’TOP mRNAs. (**A and B**). Extracts of HEK293T cells that were transfected with empty vector (EV), FLAG-tagged wild type LARP1 (WT) or FLAG-LARP1 double-mutant (R840E/Y883A), were immunoprecipitated with anti-eIF4G antibody. Inputs were analyzed by Western blot (**A**) and eIF4G-IPs were analyzed for TOP mRNA abundance by RT-ddPCR (**B**). Data were normalized to input mRNA levels. Three biological replicates were performed and error bars denote propagated standard deviation. **DOI:** http://dx.doi.org/10.7554/eLife.24146.016 10.7554/eLife.24146.017Figure 3—source data 1.Data analysis for eIF4G IPs.**DOI:** http://dx.doi.org/10.7554/eLife.24146.017

To investigate the mechanism by which LARP1 hinders the assembly of the eIF4F complex on TOP mRNAs we generated mutations in two key amino acids (R840 and Y883) that play pivotal roles in TOP mRNA binding (R840 and Y883) and in recognizing the m^7^Gppp cap structure (Y883) (Lahr et al., 2015). We compared the effects of overexpression of full-length wild type LARP1 on the assembly of the eIF4F complex on TOP (RPS6 and RPL32) and non-TOP (β-actin) mRNAs with that of the full-length R840E/Y883A double mutant. Expression of wild type LARP1 hinders the binding of endogenous eIF4G to both RPS6 and RPL32 mRNAs, but not to β-actin mRNA, as determined by RNA-immunoprecipitation/RT-ddPCR (Figure 3). This finding is consistent with the model that LARP1 binds to the cap and 5’TOP sequence of TOP mRNAs, thus selectively inhibiting eIF4F assembly on this class of transcripts. Perhaps more importantly, expression of the LARP1 R840E/Y883A double mutant does not inhibit eIF4G association with TOP mRNAs (Figure 3), indicating that cap- and TOP-binding is essential for LARP1-mediated displacement of the eIF4F complex from TOP mRNAs. Taken together with data demonstrating mTORC1 inhibition decreases the association of eIF4G with TOP transcripts (Fonseca et al., 2015), these data suggest that mTORC1 blocks the inhibitory function of LARP1.

The data presented in this study provide important structural insights into the molecular mechanism of LARP1, a specific 5’TOP motif-binding protein. LARP1 plays a fundamental role in the specialized recognition of the m^7^GpppC motif, a unique feature of TOP mRNAs that encode all the core protein components of the ribosome and the translation apparatus. Cap-binding proteins display a variety of mRNA regulatory functions (Topisirovic et al., 2011). LARP1 regulates translation and stability of this class of mRNAs through specialized 5’TOP motif (Fonseca et al., 2015; Lahr et al., 2015; Aoki et al., 2013) and cap recognition. eIF4E and its binding partner, eIF4G, have been previously linked to the control of TOP mRNA translation (Thoreen et al., 2012; Hsieh et al., 2012). Our work demonstrates that LARP1 displaces eIF4E from capped TOP mRNAs, thereby preventing the assembly of the eIF4F complex required for translation initiation. These findings reveal a previously unrecognized mTORC1-regulated dynamic interplay between LARP1 and the eIF4F complex, whereby eIF4F stimulates TOP mRNA translation while LARP1 represses it. Notably, LARP1 exhibits higher specificity and affinity for TOP mRNAs than eIF4E does, suggesting it may function as the selective factor for TOP mRNA translation regulation. These findings corroborate our earlier report (Fonseca et al., 2015) that LARP1 functions as a repressor of TOP mRNA translation: overexpression of LARP1 in mammalian cells leads to reduced TOP mRNA translation, as inferred by the accumulation of TOP mRNAs in subpolysomal fractions. Conversely, depletion of LARP1 protein from mammalian cells leads to an accumulation TOP messages in heavy polysomal fractions (Fonseca et al., 2015), indicating that LARP1 represses TOP mRNA translation. Importantly, LARP1 is essential for the ability of allosteric (rapamycin) and active-site (Torin1) mTOR inhibitors to effectively suppress TOP mRNA translation (Fonseca et al., 2015), consistent with LARP1 functioning downstream of mTORC1.

It is noteworthy that whilst LARP1 is shown here to repress TOP translation, in cancer cells LARP1 has been shown to have a positive effect on overall protein synthesis (Burrows et al., 2010) and bind many mRNA targets, including those encoding oncogenes (Hopkins et al., 2016; Mura et al., 2015), in addition to TOP mRNAs (Aoki et al., 2013; Fonseca et al., 2015; Lahr et al., 2015; Tcherkezian et al., 2014). This suggests that LARP1, while repressing TOP translation, can activate the translation of other targets, although the mechanism for the latter has yet to be elucidated.

In conclusion, our findings elucidate a novel mechanism of translation control whereby LARP1 competes with eIF4E for binding the cap of TOP mRNAs, effectively preventing eIF4F complex assembly on TOP mRNAs and ultimately precluding the translation of this class of mRNAs. Our studies now establish LARP1 as a *bona fide* cap-binding protein and the long-sought regulator of TOP mRNA translation.

## Materials and methods

### Protein expression and purification

The DM15 construct (aa 796–946) from LARP1a (BC001460.2) was expressed, purified, concentrated, and stored as previously reported (Lahr et al., 2015). Point mutations in DM15 were generated by site directed mutagenesis, and expressed and purified similarly. eIF4E (BC043226) was cloned into a modified pET28a vector expressing an N-terminal His_6_-MBP tag followed by a TEV protease site. pET28-eIF4E was transformed into BL21(DE3) cells and plated on LB supplemented with kanamycin. Cells from one ~90% confluent plate were transferred to 500 mL ZY auto-induction media for 3 hr at 37°C and then flasks were transferred overnight to 18°C (Studier, 2005). Cells were pelleted and flash frozen in liquid nitrogen for storage at −80°C.

Cells expressing eIF4E were resuspended in lysis buffer (50 mM Tris, pH 7.4, 500 mM NaCl, 1 mM β-mercaptoethanol, 20 mM imidazole, 10% glycerol) and homogenized. Lysed cells were clarified by centrifugation and the soluble fraction was incubated with Ni-NTA nickel resin (ThermoFisher Scientific (Waltham, MA), cat. no. 88221). Resin was washed three times with wash buffer (50 mM Tris, pH 7.4, 500 mM NaCl, 1 mM β-mercaptoethanol, 35 mM imidazole, 10% glycerol) and eluted in elution buffer (50 mM Tris, pH 7.4, 500 mM NaCl, 1 mM β-mercaptoethanol, 250 mM imidazole, 10% glycerol). His_6_-MBP-eIF4E was TEV-cleaved overnight in Q/SP start buffer (50 mM Tris, pH 8.0, 150 mM NaCl, 5 mM β-mercaptoethanol, 10% glycerol). Cleaved eIF4E was separated from contaminating MBP and TEV by tandem anion and cation exchange chromatography.

### Crystallization and structure solution

#### m^7^GTP-DM15 co-crystal

DM15 was concentrated to 30 mg/mL (1620 µM) for crystallization in DM15 crystallization buffer (50 mM HEPES, pH 7.0, 50 mM NaCl, 2 mM DTT). m^7^GTP (Sigma-Aldrich (St. Louis, MO), cat. no. M6133) was resuspended at a concentration of 4 mM in the same buffer. DM15 and m^7^GTP were incubated at a 1:1.3 ratio (850 µM DM15: 1100 µM m^7^GTP) in 50 mM HEPES, pH 7.0, 50 mM NaCl, 2 mM DTT at room temperature for 30 min. Crystals were grown at room temperature by sitting drop vapor diffusion using a 1:1 ratio of protein to mother liquor solution of 0.1 M HEPES, pH 7.5%, 10% PEG 6000. Crystals were stabilized in the same buffer +5% ethylene glycol and frozen in liquid nitrogen.

X-ray diffraction data were collected using the home source at the Department of Structural Biology (University of Pittsburgh) using a FR-E rotating anode generator and an R-AXIS HTC IP. Data were indexed, merged, and scaled using HKL-2000 (Otwinowski and Minor, 1997). Initial phases were generated with Phaser using the *apo* DM15 structure (PDBID: 5C0V) as the search model (McCoy et al., 2007). Iterative model building and refinement were performed with COOT and PHENIX, respectively, utilizing TLS, B-factor and positional refinement, and simulated annealing (Adams et al., 2010; Emsley et al., 2010). Ligand restraints were generated using eLBOW (Moriarty et al., 2009). A simulated annealing composite omit map confirmed amino acid positions of the final model.

#### RPS6-DM15 co-crystal

DM15 was concentrated to 20 mg/mL for crystallization in 50 mM HEPES, pH 7.0, 2 mM DTT. RPS6 RNA oligonucleotide was synthesized by IDT (5’ CUUUUCCG 3’) and was resuspended in 20 mM sodium cacodylate (Hampton Research Aliso Viejo, CA), pH 6.5. DM15 and RNA were incubated at a 1:1.1 ratio of DM15:RNA with a final concentration of 10 mg/mL DM15 in the presence of 4X binding buffer (100 mM NaCl, 4 mM DTT). This usually resulted in precipitation, so 4 M NaCl was added 1 µL at a time and mixed until the solution cleared (~100 mM NaCl final). Crystals were grown by vapor diffusion at room temperature by sitting drop using 1:1 ratio of protein-RNA complex to mother liquor solution of 5 mM MgCl_2_, 50 mM HEPES, pH 7.0, 25% PEG MME 550 (Hampton Research Natrix HR2-116 #31). Crystals were looped directly from the drop and frozen in liquid nitrogen.

X-ray diffraction data were collected at the home source at the Department of Structural Biology using a FR-E rotating anode generator and an R-AXIS IV++ IP. Data were indexed, merged, and scaled using HKL-2000 (Otwinowski and Minor, 1997). Initial phases were generated with Phaser using the *apo* DM15 structure (PDBID: 5C0V) as the search model (McCoy et al., 2007; Lahr et al., 2015). Iterative model building and refinement were performed with COOT and PHENIX, respectively, utilizing simulated annealing, grouped B-factors and rigid-body refinement (Adams et al., 2010; Emsley et al., 2010). A simulated annealing composite omit map confirmed amino acid and nucleotide positions of the final model.

#### m^7^GpppC-DM15 co-crystal

DM15 was concentrated to 26 mg/mL (1405 µM) for crystallization in DM15 crystallization buffer (50 mM HEPES, pH 7.0, 100 mM NaCl, 2 mM DTT). m^7^GpppC (40 mM stock generously provided by Utz Fischer and Nahum Sonenberg) was diluted at a concentration of 4 mM in the same buffer. DM15 and m7GpppC were incubated at a 1:1.3 ratio (850 µM DM15: 1100 µM m^7^GpppC) in 50 mM HEPES, pH 7.0, 100 mM NaCl, 2 mM DTT at room temperature for 30 min. Protein/RNA complex will precipitate quickly at 4°C, so all reagents must be kept at room temperature. Crystals were grown at room temperature by sitting drop vapor diffusion using a 1:1 ratio of protein to mother liquor solution of 0.08 M Magnesium acetate tetrahydrate, 0.05 M Sodium cacodylate trihydrate pH 6.5, 30% w/v PEG 4000 (#25 Index-116). Crystals were looped directly from the drop and frozen in liquid nitrogen.

X-ray diffraction data were collected using the home source at the Department of Structural Biology (University of Pittsburgh) using a FR-E rotating anode generator and an R-AXIS HTC IP. Data were indexed, merged, and scaled using HKL-2000 (Otwinowski and Minor, 1997). Initial phases were generated with Phaser using the apo DM15 structure (PDBID: 5C0V) as the search model (McCoy et al., 2007). Iterative model building and refinement were performed with COOT and PHENIX, respectively, utilizing TLS, B-factor and positional refinement, and simulated annealing (Adams et al., 2010; Emsley et al., 2010). Ligand restraints were generated using eLBOW (Moriarty et al., 2009). A composite omit map confirmed amino acid positions of the final model.

All figures containing refined models were generated with the PyMOL Molecular Graphics System (Schrödinger, LLC. (New York, NY)).

### RNA synthesis for biochemical assays

RNAs were in vitro transcribed and purified as described previously (Lahr et al., 2015). For studying the un-capped RNAs, transcribed RNAs were treated with alkaline phosphatase (Roche Life Sciences (Indianapolis, IN), cat. no. M183A) and 5’ end-radiolabeled with [γ-^32^P]-ATP. To generate capped RNAs, the 5’ triphosphate required for the capping reaction was regenerated in TOP RNAs by incubation of 50 nM RNA with T4 PNK in PNK buffer A (ThermoFisher Scientific cat. no. EK0032) with 3 mM ATP for 20 min at 37°C followed by addition of 5 units of nucleoside monophosphate kinase (Roche Life Sciences, cat. no. 10107948001). RNA was purified by phenol-chloroform extraction, MicroSpin G-25 desalting columns (GE Healthcare Life Sciences (Marlborough, MA), cat. no. 27-5325-01), and ethanol precipitation. RNAs with a 5’ triphosphate were subsequently capped and radiolabeled using vaccinia capping enzyme (NEB (Ipswich, MA), cat. no. M2080S) and [α-^32^P]-GTP or GTP according to the manufacturer’s protocol.

### Electrophoretic mobility shift assays (EMSAs)

EMSAs were performed and imaged as reported previously using the same amount of RNA (≤200 pM) regardless of labeling efficiency (Lahr et al., 2015). All RNAs were snap-cooled by heating at 95°C in 1X binding buffer for 1 min and immediately transferred to ice for 20 min. Replicates were quantified using ImageQuant TL (GE Healthcare Life Sciences) and graphed using KaleidaGraph (Synergy Software) as previously reported (Lahr et al., 2015).

### RNA competition assays

DM15 was prepared as 5X protein stocks in protein dilution buffer (50 mM Tris, pH 7.5, 250 mM NaCl, 25% glycerol, 2 mM DTT) and prebound to m^7^GpppG-RPS6 for 30 min on ice in 8 µL reactions. 2 µL of 5X cold competitor RNA was titrated for final reaction conditions of 20 mM Tris, pH 8, 150 mM NaCl, 10% glycerol, 1 mM DTT, 0.5 µg BSA, 0.5 µg tRNA. Competitions were incubated for an additional 30 min on ice before loading and analyzing on native gels using the same methods as the EMSAs.

### eIF4E and DM15 competition assays

DM15 or eIF4E 5X protein dilutions were prepared in protein dilution buffer and pre-bound to RNA substrates for 30 min on ice in 8 µL reactions. 2 µL of 5X competitor protein was titrated for final reaction conditions of 20 mM Tris, pH 8, 150 mM NaCl, 10% glycerol, 1 mM DTT, 0.5 µg BSA, 0.5 µg tRNA. Competitions were incubated for an additional 30 min on ice before and analyzing on native gels using the same methods as the EMSAs.

### RNA sequences

The RNA oligonucleotides used for crystallization and biochemical assays were as follows:

RPS6 8mer (IDT (Coralville, IA)): 5’ CUUUUCCG 3’

RPS6 42mer (Sigma-Aldrich):

5’ CCUCUUUUCCGUGGCGCCUCGGAGGCGUUCAGCUGCUUCAAG 3’.

G-RPS6 was T7-transcribed from a DNA template to generate the RNA sequence:

5’ GCCUCUUUUCCGUGGCGCCUCGGAGGCGUUCAGCUGCUUCAAG 3’.

RPL32 was T7-transcribed from a DNA template designed with a self-cleaving 5’ hammerhead ribozyme to generate a 5’ C, resulting in the final RNA sequence:

5’ CUCUCUUCCUCGGCGCUGCCUACGGAGGUGGCAGCCAUCUCC 3’.

#### Thermal shift assays

100 µM protein was incubated with a saturating amount of GTP, ATP or m^7^GTP (1 mM) in 1X EMSA binding buffer and 1X SYPRO orange (Promega (Madison, WI), cat. no. s5692) in 50 µL reactions at room temperature for 20 min. Protein unfolding was measured by monitoring fluorescence of SYPRO orange at 570 nm during a temperature ramp from (25–95°C). Fluorescence data were analyzed using R-studio and the Boltzmann model to calculate the melting temperature. Three biological replicates were performed for each experiment and averaged. The ∆T_m_ was determined to be the difference between the T_m_ of the protein alone and T_m_ of the protein in the presence of the ligand.

### Sequence alignments

Sequences were aligned with BLAST (Altschul et al., 1997) and frequency plots were generated with WebLogo (Crooks et al., 2004).

### Cell culture

HEK293T cells (human embryo kidney 293 transformed with the simian virus 40 large T antigen, cat. no. CRL11268^TM^ and lot no. 59521234), used in Figure 3, were purchased from ATCC (Manassas, VA) in January 2012, expanded and stored in liquid nitrogen. Low passage cells were thawed and tested for mycoplasma infection every 6 months. Cells scored negative for mycoplasma infection in every instance. Cells were maintained in DMEM (Hyclone/GE Healthcare, cat. no. SH30022.01) supplemented with 10% (v/v) fetal bovine serum (Sigma-Aldrich, cat. no. F1051-500ml) and 1% (v/v) penicillin/streptomycin in a humidified incubator at 37°C and 5% (v/v) CO_2_.

### Cell lysis, protein extraction, and RNA immunoprecipitation

RNA immunoprecipitation was carried out as follows: HEK293T cells were seeded at approximately 15 million cells per plate on 15 cm plates and propagated in DMEM (Hyclone/GE Healthcare Life Sciences, cat. no. SH30022.01) supplemented with 10% (v/v) fetal bovine serum (Sigma-Aldrich, cat. no. F1051-500ml) and 1% (v/v) penicillin/streptomycin in a humidified incubator at 37°C and 5% (v/v) CO_2_. Approximately 24 hr after seeding, cells were transfected with various amounts of pCMV5 empty vector or pCMV6-human LARP1 (1019 aa) C-terminally myc- and FLAG-tagged. Typically, 4 to 8 μg of plasmid DNA were used for transfecting HEK293T cells in a 15 cm dish. Cells were transfected with lipofectamine 2000 reagent (Invitrogen (Carlsbad, CA), cat. no. 11668–019) as per manufacturer’s instructions. Plasmids were expressed for 24 hr prior to lysis in CHAPS extraction buffer containing 40 mM HEPES (pH 7.5 at room temperature), 0.3 (w/v) CHAPS zwitterionic detergent, 120 mM NaCl, 1 mM EDTA, 10 mM sodium pyrophosphate, 10 mM β-glycerophosphate, 50 mM sodium fluoride (Ser/Thr phosphatase inhibitor), 1.5 mM sodium orthovanadate (Tyr phosphatase inhibitor), 1 mM DTT, complete EDTA-free protease inhibitors mixture tablets (Roche Life Sciences, cat. no. 04693132001). Briefly, cells were washed once with 10 mL ice-cold phosphate buffer saline followed by 3 mL of CHAPS lysis buffer. Cells were incubated with lysis buffer for 45 min and then scraped and collected in a microfuge tube. Lysates were pre-cleared by centrifugation at 21,000 x g for 1 min at 4°C. Supernatant was collected and 1 mL lysate was used for immunoprecipitation with 10 μL of eIF4G antibody (Cell Signaling Technologies (Danvers, MA), cat. no. 2469) or 5 μL of FLAG antibody (Sigma-Aldrich, cat. no. F1804-200 μg). Lysates were incubated with antibody for 1 hr at 4°C mixing end-over-end. Twenty microliters of bed-volume of protein G-conjugated magnetic dynabeads (Life Technologies (Carlsbad, CA), cat. no. 10004D) were added to the lysate/antibody mixture and incubated for an additional 30 min at 4°C with mixing end-over-end. Beads were collected with a magnetic rack and washed 3 times with 1 vol (1 mL) of CHAPS extraction buffer described above. RNA was then extracted with RNAzol RT solution (Sigma-Aldrich, cat. no. R4533-500 mL). 1 mL of RNAzol was added to the beads/antibody/protein/RNA mixture, followed by the addition of 1 mL of CHAPS lysis buffer. Samples were vortexed for 15 s and incubated at room temperature for 15 min, and then centrifuged at maximal speed on a table-top centrifuge for 15 min at 4°C. The aqueous phase was collected (900 μL) and ethanol precipitated. The total RNA pellet was air-dried and resuspended in 100 μL RNAse-free water (Sigma-Aldrich, cat. no. W4502-1L) for inputs and in 10 μL RNAse-free water for immunoprecipitates.

### Reverse transcription-digital droplet PCR (RT-ddPCR)

Reverse-transcription reactions were carried out using the iScript advanced cDNA synthesis kit (Bio-Rad (Hercules, CA), cat. no. 172–5038) as per manufacturer’s protocol with modifications. Briefly, 4 μL of 5X advanced reaction mix were added to 1 μL advanced reverse transcriptase and 10 μL of RNA template supplemented with RNAse-free water to a final volume of 20 μL. The reaction mixture was incubated at 46°C for 1 hr followed by 95°C for 1 min. The cDNA reaction was then diluted 1000X for eIF4G IPs and 5000X for (LARP1 IPs) in RNAse free water prior to analysis by digital droplet PCR (ddPCR). Each ddPCR reaction was carried out by adding 10 μL QX200 ddPCR EvaGreen Supermix (Bio-Rad, cat. no. 186–4034), 0.2 μL of each primer (forward and reverse) at a stock concentration of 10 μM, 8 μL of diluted cDNA, and 1.6 μL RNAse-free water to a final volume of 20 μL reaction. The reaction mixtures were transferred to DG8 Cartridges for the QX100/QX200 Droplet Generator (Bio-Rad, cat. no.186–4008) and 70 μL Droplet Generation Oil for EvaGreen were added (Bio-Rad, cat. no.186–4006). Samples were processed on the droplet generator and then transferred to 96-well ddPCR plates followed by sealing with aluminum foil. Thermal cycling was run using the following conditions: 95°C for 5 min, 95°C for 30 s, ramp down 2°C/s until it temperature reaches 62°C, then ramp up temperature to 95°C; repeat this cycle 45 times. Samples were then cooled to 4°C for 5 min, heated up to 95°C 5 min, and lastly held at 12°C indefinitely. Samples were analyzed on Bio-Rad QX200 droplet plate reader.

## Additional files

### Major datasets

The following datasets were generated:

Berman AJ,Lahr RM,Al-Ashtal HA,2017,DM15-RNA cocrystal,http://www.rcsb.org/pdb/explore/explore.do?structureId=5V7C,Publicly available at the RCSB Protein Data Bank (accession no: 5V7C)

Berman AJ,Lahr RM,Al-Ashtal HA,2017,DM15-m7GTP cocrystal,http://www.rcsb.org/pdb/explore/explore.do?structureId=5V4R,Publicly available at the RCSB Protein Data Bank (accession no: 5V4R)

Berman AJ,Lahr RM,Al-Ashtal HA,2017,DM15-m7GpppC cocrystal,http://www.rcsb.org/pdb/explore/explore.do?structureId=5V87,Publicly available at the RCSB Protein Data Bank (accession no: 5V87)
