# Supplementary material for: La-related protein 1 (LARP1) binds the mRNA cap, blocking eIF4F assembly on TOP mRNAs
Source: eLife. 2017 Apr 7;6:e24146. doi: 10.7554/eLife.24146 (PMC5419741; doi:10.7554/eLife.24146)
Supplement: Related texts

# Article commentary: Decision letter

**DOI:** 10.7554/eLife.24146.024

**Affiliations:** Johns Hopkins University, United States

In the interests of transparency, eLife includes the editorial decision letter and accompanying author responses. A lightly edited version of the letter sent to the authors after peer review is shown, indicating the most substantive concerns; minor comments are not usually included.

Thank you for submitting your article "La-related protein 1 (LARP1) binds the mRNA cap, blocking eIF4F assembly on TOP mRNAs" for consideration by *eLife*. Your article has been favorably evaluated by John Kuriyan (Senior Editor) and three reviewers, one of whom is a member of our Board of Reviewing Editors. The following individual involved in review of your submission has agreed to reveal their identity: Alan G Hinnebusch (Reviewer #3).

The reviewers have discussed the reviews with one another and the Reviewing Editor has drafted this decision to help you prepare a revised submission.

Summary:

This is an elegant study that reveals a new and unanticipated mechanism by which LARP1 regulates translation of TOP mRNAs by competing with translation initiation factors for binding to the mRNA CAP. Efficient translation of these mRNAs in nutrient-replete cells depends on mTORC1. LARP1 has been implicated recently as a target of mTORC1 and was previously shown by this group to bind the 5'TOP motif on TOP mRNAs via the DM15 region. In the present manuscript, the authors discover that DM15 also has cap-binding activity by noting fortuitous interactions in their crystal structure of the human LARP1 DM15 domain bound to an RNA fragment. In the crystal, a guanine nucleotide from one LARP-RNA complex bound to a site in a neighboring LARP1 protein that the authors cleverly surmised might mimic the guanine cap of the remaining RNA fragment. This hypothesis is supported by a crystal structure of LARP1 bound to an mRNA CAP analogue, in vitro binding studies demonstrating a preference for capped TOP mRNA and competition studies showing that LARP1 DM15 competes for mRNA binding to the initiation factor, eIF4E. LARP1 is thus a novel cap-binding protein that couples recognition of the m^7^G cap with that of the 5'-TOP sequences, particularly a conserved 5'-terminal C residue. Consistent with these data, ectopic expression of wild type, but not mutant, LARP1 reduces co-IP of two native TOP mRNAs with eIF4G, but not the non-TOP actin mRNA.

The three reviewers were enthusiastic about this exciting addition to our understanding of the mechanism by which mTORC1, which regulates LARP1 activity, enhances TOP mRNA translation and thereby stimulates growth. By revealing a previously unknown activity of LARP1, the work represents a conceptual advance in understanding how translation of components of the protein synthesis machinery is regulated.

Essential revisions:

The manuscript is somewhat tersely written and would thus benefit overall from more detailed explanations of the authors' logic and relation of their results to prior work. The Introduction needs more details about exactly what has already been shown for LARP1 in terms of inhibiting the translation of TOP mRNAs and mediating the stimulatory effects of mTORC1 on TOP mRNA translation in extracts or cells. The authors should also do a better job of explaining to the general readership how the interactions in the crystal lattice led them to speculate that they were mimicking binding of a capped RNA and how the contacts they observe co-specify binding to the m^7^GpppC cap. The authors should also cite and discuss results from Fonseca et al., 2015, JBC that support their new model, including the finding that LARP1 overexpression led to a shift of mRNAs encoding ribosomal proteins towards lighter fractions in polysome density gradients, while knocking down LARP1 caused the re-distribution of TOP mRNAs from light to heavy polysomal fractions.

# Reply: Author response

**DOI:** 10.7554/eLife.24146.025

*Essential revisions:*

*The manuscript is somewhat tersely written and would thus benefit overall from more detailed explanations of the authors' logic and relation of their results to prior work. The Introduction needs more details about exactly what has already been shown for LARP1 in terms of inhibiting the translation of TOP mRNAs and mediating the stimulatory effects of mTORC1 on TOP mRNA translation in extracts or cells. The authors should also do a better job of explaining to the general readership how the interactions in the crystal lattice led them to speculate that they were mimicking binding of a capped RNA and how the contacts they observe co-specify binding to the m^7^GpppC cap. The authors should also cite and discuss results from Fonseca et al., 2015, JBC that support their new model, including the finding that LARP1 overexpression led to a shift of mRNAs encoding ribosomal proteins towards lighter fractions in polysome density gradients, while knocking down LARP1 caused the re-distribution of TOP mRNAs from light to heavy polysomal fractions.*

We have significantly lengthened the manuscript. We include a detailed explanation of the logic used to deduce that LARP1 is a cap-binding protein. We have also expanded the Introduction to more adequately address the contributions of Fonseca et al., 2015 to set the stage for understanding LARP1 in TOP mRNA translation regulation. Finally, we have determined the crystal structure of DM15 bound to m^7^GpppC, which has been included in Figure 1 and in the relevant Figure 1 figure supplements.
